# Investigation of factors that may potentially affect anxiety in patients undergoing esophagogastroduodenoscopy and evaluation of sedation effect

**DOI:** 10.1097/MD.0000000000037801

**Published:** 2024-04-12

**Authors:** Burak Uçaner, Mehmet Zeki Buldanli, Şebnem Çimen, Mehmet Sabri Çiftçi, Mehmet Mert Demircioğlu, Ela Erten, Oğuz Hançerlioğullari

**Affiliations:** a Department of General Surgery, University of Health Sciences, Gülhane Training and Research Hospital, Ankara, Turkey; b Department of General Surgery, Sincan Training and Research Hospital, Ankara, Turkey; c Department of Anaesthesiology, University of Health Sciences, Gülhane Training and Research Hospital, Ankara, Turkey.

**Keywords:** Beck anxiety inventory, endoscopy, esophagogastroduodenoscopy, sedation

## Abstract

**Background:**

As with any other invasive procedure, esophagogastroduodenoscopy (EGD) may lead to considerable anxiety in patients. This study aimed to investigate and compare the effects of sedated and non-sedated procedures on anxiety in patients undergoing EGD and to better recognize patient groups at risk for anxiety.

**Methods:**

In this prospective and 2-armed designed study, demographic data, including age, gender, comorbidities, height, weight, body mass index (BMI), and educational background, were collected. In this study, the Beck Anxiety Inventory (BAI) was administered to each patient before they were brought to the endoscopy unit. Subsequently, each patient who underwent EGD was telephoned on the seventh day after the procedure and the BAI was administered a second time.

**Results:**

Women population had higher pre-EGD and post-EGD BAI scores compared to men. No significant correlation was observed between educational background and BAI scores. Possible correlations between age, BMI, Charlson comorbidity index (CCI), and BAI scores were examined. There was a weak correlation between BMI and pre-EGD and post-EGD BAI scores. A strong and positive correlation was observed between the pre-EGD BAI score and post-EGD BAI and difference in BAI scores between groups (ΔBAI).

**Conclusion:**

Endoscopic procedures may cause anxiety in patients as with all other invasive procedures. Patients’ compliance with the procedure and having a lower level of anxiety are very significant for diagnostic and, if necessary, therapeutic success. In this study, the patient gender was evaluated as a predictor of anxiety level, whereas educational background was not a predictor.

## 1. Introduction

Currently, esophagogastroduodenoscopy (EGD) is one of the best diagnostic methods used in the evaluation of the upper gastrointestinal tract.^[1]^ With EGD, as well as recognition of the lesion, biopsy of the lesion and therapeutic procedures in appropriate patients are also possible.^[2]^ However, despite all these advantages, EGD is a painful and uncomfortable procedure, making it challenging for both the endoscopist and the patient. Like any other invasive procedure, EGD can induce significant anxiety in patients. In the study reported by Sargin et al, a significant relationship was found between pre-procedure anxiety levels and sedoanalgesia in patients undergoing upper GI endoscopy.^[3]^ Various anxiety scales are utilized to measure the level of anxiety in patients who undergo EGD. One of the most widely used of these scales is the Beck Anxiety Inventory (BAI).^[4,5]^

Anxiety is a common occurrence in patients undergoing interventional medical procedures, and therefore the demand for sedoanalgesia in endoscopic procedures has increased. However, it has been reported in various studies that endoscopic procedures, even with sedation, have a severe anxiety effect on patients.^[6–8]^ When looking at pre-procedure anxiety levels in the study reported by Chung et al; Minimal (Beck scores 0–7) in 65% of patients, Mild (Beck scores 9–15) in 26%, Moderate (Beck scores 16–25) in 8%, and Severe (Beck scores > 26) in 2% was reported as.^[8]^ Anxiety before and after the procedure adversely affects patients’ compliance with treatment and quality of life. In several studies, various methods, including informing patients and playing music before the procedure, have been attempted to reduce anxiety in patients.^[9–11]^ Further understanding of anxiety levels and identifying potential risk groups is crucial for optimizing the effectiveness of these methods.

When the previous studies on this subject are examined, we recognize that; There is no clear consensus on the factors affecting anxiety in patients undergoing upper GI endoscopy; Discussions on this issue still continue. The purpose of this study; To investigate the effect of sedation on patient anxiety in patients undergoing upper GI endoscopy and to better recognize patient groups at risk for anxiety.

## 2. Material and methods

### 2.1. Study population and recruitment

Ethical approvals were obtained from the Ethics Committee of Gülhane Training and Research Hospital (approval no: 2023/111). The prospectively and 2-armed designed study included 310 patients between the ages of 20 and 78 years who underwent EGD in the General Surgery Endoscopy unit within the 6-month period between July 2023 and December 2023. A written informed consent was obtained from all patients included in the study. Patients aged 18 and above, willing to participate, and scheduled for EGD were included. Patients who had previously undergone EGD, were under 18 years of age, over 80 years of age, and who were unwilling to participate in the study were excluded. Other exclusion criteria of the study so that the results of the study can be evaluated more objectively and the patient population is more homogeneous; according to the anamnesis, physical and psychological examination findings and national health system data taken from the patient on the day of the procedure; the patient had a history of psychiatric disease, the patient was using anxiolytic medication, and the patient had symptoms of dementia. In the study; before being taken to the endoscopy room, each patient was asked Beck Anxiety Scale (BAI) questions one by one by the endoscopist, and the patients were asked to score from 0 to 3 for each question. Demographic data of the patients, such as age, gender, comorbidities, height, weight, body mass index (BMI) and educational status, were collected. Before the procedure, the anesthesiologist explained the sedoanalgesia procedure to the patients and the side effects associated with the procedure, and written and verbal informed consent was obtained from the patients. Then, the procedure was performed with or without sedation, depending on the patient decision. The patients’ anesthesia type decision was not changed in any way, and routine procedures were followed.

Esophagogastroduodenoscopies performed with sedation and without sedation were grouped. Pre- and post-procedure patient anxiety was investigated with the BAI, and pre- and post-procedure scoring results were compared. The 2 patient groups were also compared within the groups.

### 2.2. Beck anxiety inventory

In the inventory, patients were asked a 21-item questionnaire with answers ranging from “not at all” (0 points) to “severely” (3 points). Scores were obtained ranging from 0 to 63 points, and the data of the questionnaire were recorded. A BAI score of 0 to 7 represents minimal anxiety, 8 to 15 represents mild anxiety, 16 to 25 represents moderate anxiety, and 26 to 63 represents severe anxiety.

In this study, the BAI was administered to each patient before they were brought to the endoscopy unit. Subsequently, each patient who underwent EGD was telephoned on the 7th day after the procedure and the BAI was administered a second time.

### 2.3. EGD procedure with sedoanalgesia

After appropriate preparation for the procedure and fasting for 8 hours, the patients who came to the unit were administered intravenous (iv) cannulation and endoscopy mouthpiece after pre-procedure and pre-assessment. After the patients were taken to the procedure room, noninvasive blood pressure, pulse oximetry, and electrocardiogram monitoring were performed, and 10% lidocaine pump spray was applied to the oropharynx. Nasal oxygen was given at 2 lt/min. The procedure was then started by giving 1 mg/kg iv propofol to the patients. During the procedure, peripheral oxygen saturation below 92% was considered hypoxia, and a pulse rate below 60/min was considered bradycardia. In general, a loading dose of 40 mg to 50 mg of propofol was given with smaller bolus loads (10–20 mg) to maintain sedation, with a typical total dose of 100 mg to 300 mg. Continuous infusions of 100 mg/h to 200 mg/h were also used as needed. The depth of sedation was determined using “The Modified Observer Assessment of Alertness and Sedation (MOAA/S).” The average duration of the EGD procedure performed on patients was 20 (10–35) minutes. The patient was moved to the recovery room after the application of EGD.

### 2.4. EGD procedure without sedoanalgesia

After appropriate preparation for the procedure and fasting for 8 hours, the patients who came to the unit were administered intravenous (iv) cannulation and endoscopy mouthpiece after pre-procedure and pre-assessment. After the patients were taken to the procedure room, noninvasive blood pressure, pulse oximetry, and electrocardiogram monitoring were performed, and 10% lidocaine pump spray was applied to the oropharynx. Nasal oxygen was given at 2 lt/min. The average duration of the EGD procedure performed on patients was 10 (5–15) minutes. The patient was moved to the recovery room after the application of EGD.

### 2.5. Statistical analysis

Statistical analyses were performed with SPSS version 22.0 software. Histograms, probability graphs, and “Shapiro–Wilk test” were used in distribution analysis. Normally distributed continuous variables were expressed as mean ± standard deviation, non-normally distributed continuous variables as median and interquartile range, and categorical data as number (percentage). The difference between pre- and post-EGD BAI scores was calculated by “Student *t* test” for normally distributed scores and by “Wilcoxon signed rank test” for non-normally distributed scores. The relationship between descriptive characteristics and pre- and post-EGD BAI scores were analyzed with “Mann–Whitney U” and “Kruskall Wallis test.” Possible correlations between age, BMI, Charlson comorbidity index (CCI), pre- and post-EGD BAI scores were evaluated with “Spearman test.” “*P* value” under “.05” was considered significant in the analyses.

## 3. Results

The mean age of the 310 patients included in the study was 50.5 ± 16.1 years (20–78 years). 60.3% of the cases were female. The male/female ratio was 1.5/1. The educational background was primary education in about half of the patients (primary school 46.8%, secondary school 6.5%). The most common reasons for presentation were dyspeptic complaints (45.5%) and malignancy screening (25.8%). Endoscopic evaluation was performed under sedation in 83.2% of cases. According to the BAI scores evaluated in the pre-EGD, 59.7% of the patients had no anxiety or minimal anxiety, 14.8% had mild anxiety, 13.9% had moderate anxiety, and 11.6% had severe anxiety. These rates were 70.6%, 19.0%, 9%, and 1.3%, respectively, post-EGD.

**Figure 1.**
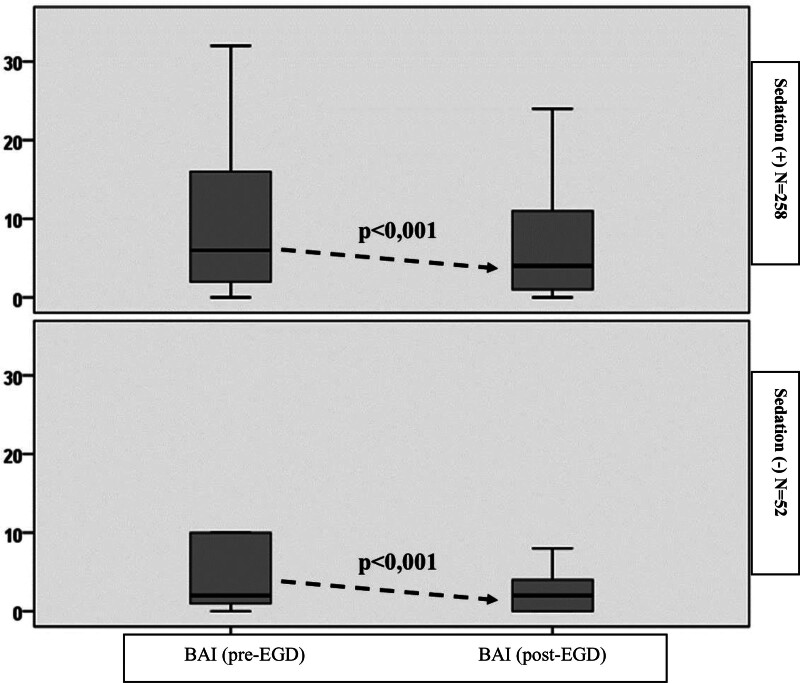
Intragroup analysis of Beck anxiety inventory scores in EGDwS and EGDwNS groups. EGDwS = esophagogastroduodenoscopies performed with sedation, EGDwNS = esophagogastroduodenoscopies performed without sedation.

Considering all cases together, it was observed that the BAI scores showed a significant decrease post-EGD (8.9 ± 9.6 vs 5.9 ± 6.7, *P* < .001). Similarly, a significant decrease was observed in post-EGD BAI scores in sedated (9.1 ± 9.6 vs 6.6 ± 7.1, *P* < .001) and non-sedated patients (7.8 ± 9.7 vs 2.3 ± 2.4, *P* < .001) (Fig. 1).

While there was no difference in pre-EGD BAI scores, post-EGD BAI scores were higher in sedated patients compared to non-sedated patients (*P* < .001). However, no difference was observed between the groups regarding the difference in BAI scores (ΔBAI) (Table 1).

**Table 1:** Comparison of sedated and non-sedated patients.

Characteristics	Group EGDwS (n = 258)	Group EGDwNS (n = 52)	*P* value
	N (%)	N (%)	
BAI (pre-EGD)*	9.1 ± 9.6	7.8 ± 9.7	.270†
Minimal anxiety (0–7)	152 (58.9)	33 (63.5)	
Mild anxiety (8–15)	37 (14.3)	9 (17.3)	
Moderate anxiety (16–25)	43 (16.7)	0	
Severe anxiety (26–63)	26 (10.1)	10 (9.2)	
BAI (post-EGD)*	6.6 ± 7.1	2.3 ± 2.4	**<.001** †
Minimal anxiety (0–7)	172 (66.7)	47 (90.4)	
Mild anxiety (8–15)	54 (20.9)	5 (9.6)	
Moderate anxiety (16–25)	28 (10.9)	0	
Severe anxiety (26–63)	4 (1.6)	0	
ΔBAI*	1 (4)	2 (9)	.188†

BAI = Beck anxiety inventory, EGD = esophagogastroduodenoscopy, ΔBAI = difference in BAI scores between groups, EGDwS = esophagogastroduodenoscopies performed with sedation, EGDwNS = esophagogastroduodenoscopies performed without sedation.

* Median (Inter Quantile Range).

† Mann–Whitney U test.

The BAI scores of the patients were analyzed according to gender, educational background, and complaints at presentation. Female patients had higher pre-EGD (*P* < .001) and post-EGD (*P* < .001) BAI scores than male patients. No significant correlation was observed between educational background and BAI scores (Table 2).

**Table 2:** Analysis of Beck anxiety inventory scores by gender and education level.

Characteristics	BAI (pre-EGD)	*P* value	BAI (post-EGD)	*P* value
	Mean ± SD		Mean ± SD	
Gender		**<.001** †		**<.001** †
Female	11.7 ± 10.1		7.8 ± 7.1	
Male	4.7 ± 6.8		3.0 ± 4.9	
Education level		.062††		.662††
Primary school	9.8 ± 9.0		5.8 ± 6.2	
Secondary school	3.8 ± 2.8		4.4 ± 3.7	
High school	6.7 ± 8.4		4.9 ± 5.3	
University	11.3 ± 12.3		7.8 ± 9.4	

BAI = Beck anxiety inventory, EGD = esophagogastroduodenoscopy.

† Mann–Whitney U test.

†† Kruskal Wallis test.

Possible correlations between age, BMI, CCI, and BAI scores were examined. There was a strong and positive correlation between age and CCI (*P* < .001). There was a weak correlation between BMI and pre-EGD BAI (*P* < .001) and post-EGD BAI (*P* < .001) scores. A strong and positive correlation was observed between the pre-EGD BAI score and post-EGD BAI (*P* < .001) and ΔBAI (*P* < .001) (Table 3).

**Table 3:** Correlation analysis between age, body mass index, Charlson comorbidity index, and Beck anxiety inventory scores.

		Age	BMI	CCI	BAI1	BAI2	ΔBAI
Age	Rho/r	-	0.037	**0.894**	−0.010	−0.029	0.066
	*P* value	-	.514	**<.001**	.862	.609	.246
BMI	Rho/r	-	-	0.027	−**0.202**	−**0.229**	0.011
	*P* value	-	-	.633	**<.001**	**<.001**	.852
CCI	Rho/r	-	-	-	0.048	0.015	0.081
	*P* value	-	-	-	.400	.794	.153
BAI1	Rho/r	-	-	-	-	**0.682**	**0.661**
	*P* value	-	-	-	-	**<.001**	**<.001**
BAI2	Rho/r	-	-	-	-	-	−0.001
	*P* value	-	-	-	-	-	.989
ΔBAI	Rho/r	-	-	-	-	-	-
	*P* value	-	-	-	-	-	-

Spearman correlation test was used in all analyses.

BAI = Beck anxiety inventory, BAI1 = pre-esophagogastroduodenoscopy BAI score, BAI2 = post-esophagogastroduodenoscopy BAI score, BMI = body mass index, CCI = Charlson comorbidity index, ΔBAI = difference in BAI scores between groups.

No procedure-related anesthetic or endoscopic complications were observed during the follow-up interval.

## 4. Discussion

Endoscopic procedures have become increasingly utilized for diagnostic and therapeutic purposes and have taken the first place among diagnostic tests for GIT pathologies. This is due to the fact that endoscopic methods provide more objective results compared to other examinations and are easily accessible and cost-effective. As with all invasive procedures, endoscopy may cause anxiety in patients. We investigated the effect of age, gender, comorbidity, educational background, and endoscopy indication on pre-procedural and post-procedural anxiety scores of patients undergoing upper gastrointestinal tract endoscopy with and without sedation.

In our study, when the patient groups who underwent upper GI endoscopy under sedation and without sedation were examined. It was observed that BAI scores did not make a significant difference before the procedure, but after the procedure, BAI scores were calculated significantly higher in the group without sedation (*P* < .001). Similar results were reported in the study conducted by Jin et al.^[12]^ In our study, significantly lower reporting of BAI scores after the procedure, we think that the higher patient comfort in procedures performed with sedoanalgesia and the fact that patients have prejudices about the procedure to be performed before the procedure affect these results.

An analysis of the patients’ BAI scores by gender and educational background revealed that the BAI scores of female patients before and after the procedure were significantly higher than those of male patients (*P* < .001). No significant difference was found between the patients regarding their educational background. In the study conducted by Sargin et al,^[13]^ the BAI score was reported to be significantly higher in female patients than in male patients. In the same study, the patients’ educational background was reported to be significant, contrary to our study. However, no significant difference was found between the patients’ educational background and anxiety in the study carried out by Salmore et al.^[14]^ Different results have been reported in studies investigating the effects of gender and educational status on patient anxiety, and debates on this issue still continue. Although the effect of female gender on anxiety was reported as a significant variable in our study, in line with the literature information; there are differences between the literature information and the results of our study regarding the educational status of the patients. We believe that the difference in the patient populations studied is effective in finding different results regarding the effect of educational status on anxiety. In the study conducted by Dessotte et al, it was reported that being a female gender creates a greater tendency to anxiety compared to male gender.^[15]^ In another study, Kinrys et al^[16]^ when comparing women with men; they reported that the risk of developing anxiety disorders throughout life is higher in women. In addition, study results reported that symptom severity, chronic course, and functional impairment were generally increased in women with anxiety disorders compared to men. However, the reasons for the increased risk of developing anxiety disorders in women are still unknown and debates on this subject continue. Evidence from several studies suggests that genetic factors and female reproductive hormones may play important roles in the expression of these sex differences.^[17,18]^

When all cases were evaluated together in our study, it was observed that the BAI scores showed a significant decrease post-EGD (*P* < .001). Similarly, there was a significant decrease in post-EGD BAI scores in sedated (*P* < .001) and non-sedated (*P* < .001) patients. Although detailed information was given to the patients by the endoscopist, we think that the decrease in the prejudices of the patients after the procedure and the decrease in anxiety after the procedure were effective in obtaining these results. Similar studies on this subject have reported findings compatible with our results.^[11,19,20]^

In our study, BAI, which was also used in previous studies on this subject,^[8]^ was preferred; when other current studies in the literature regarding anxiety level measurement are examined; it was observed that other anxiety scales were also used. Instead of using the state-trait anxiety inventory, which consists of 40 questions and used in the study of Jones et al^[6]^; we chose to use BAI, which consists of 21 questions, has a language that patients can understand more easily, and we think can be applied more easily by the endoscopist.

The study limitations were that the procedures were not performed and sedated by a single endoscopist and by a single anesthesiologist, and that the number of patients was comparatively different in the sedated and non-sedated patient groups.

## 5. Conclusions

In our study, BAI scores of the cases were analyzed according to the patients’ gender, education level and admission complaints. BAI scores of women before and after endoscopy were found to be higher than men. No significant relationship was observed between education level and BAI scores.

In conclusion, endoscopic procedures cause anxiety in patients, as with all other invasive procedures. It is very important for patients to adapt to the procedure and have a lower level of anxiety in terms of success in the treatment process. When the studies on the subject are examined, we observed that, debates still continue regarding the factors affecting anxiety. With studies with larger and more homogeneous populations, questions in the minds of clinicians will be answered in the future and controversial issues in the literature will be concluded.

## Author contributions

**Conceptualization:** Burak Uçaner, Şebnem Çimen.

**Data curation:** Burak Uçaner, Şebnem Çimen, Mehmet Sabri Çiftçi, Mehmet Mert Demircioğlu.

**Formal analysis:** Mehmet Zeki Buldanli, Mehmet Sabri Çiftçi, Ela Erten, Oğuz Hançerlioğullari.

**Funding acquisition:** Burak Uçaner, Mehmet Sabri Çiftçi.

**Investigation:** Burak Uçaner, Mehmet Zeki Buldanli, Şebnem Çimen.

**Methodology:** Burak Uçaner, Şebnem Çimen, Mehmet Sabri Çiftçi, Oğuz Hançerlioğullari.

**Project administration:** Burak Uçaner, Ela Erten, Oğuz Hançerlioğullari.

**Resources:** Mehmet Zeki Buldanli.

**Software:** Burak Uçaner, Şebnem Çimen, Mehmet Mert Demircioğlu.

**Supervision:** Burak Uçaner, Mehmet Sabri Çiftçi, Ela Erten, Oğuz Hançerlioğullari.

**Validation:** Burak Uçaner, Mehmet Sabri Çiftçi, Oğuz Hançerlioğullari.

**Visualization:** Mehmet Zeki Buldanli, Mehmet Mert Demircioğlu.

**Writing – original draft:** Burak Uçaner, Mehmet Zeki Buldanli.

**Writing – review & editing:** Burak Uçaner, Mehmet Zeki Buldanli.
